# Comparison of arterial and venous allograft bypass in chronic limb-threatening ischemia

**DOI:** 10.1371/journal.pone.0275628

**Published:** 2022-10-27

**Authors:** Dávid Garbaisz, Péter Osztrogonácz, András Mihály Boros, László Hidi, Péter Sótonyi, Zoltán Szeberin

**Affiliations:** 1 Department of Vascular and Endovascular Surgery, Heart and Vascular Center, Semmelweis University, Budapest, Hungary; 2 Department of Cardiology, Heart and Vascular Center, Semmelweis University, Budapest, Hungary; Szegedi Tudomanyegyetem, HUNGARY

## Abstract

**Introduction:**

Femoro-popliteal bypass with autologous vascular graft is a key revascularization method in chronic limb-threatening ischemia (CLTI). However, the lack of suitable autologous conduit may occur in 15–45% of the patients, necessitating the implantation of prosthetic or allogen grafts. Only little data is available on the outcome of allograft use in CLTI.

**Aims:**

Our objective were to evaluate the long term results of infrainguinal allograft bypass surgery in patients with chronic limb-threatening ischemia (CLTI) and compare the results of arterial and venous allografts.

**Methods:**

Single center, retrospective study analysing the outcomes of infrainguinal allograft bypass surgery in patients with CLTI between January 2007 and December 2017.

**Results:**

During a 11-year period, 134 infrainguinal allograft bypasses were performed for CLTI [91 males (67.9%)]. Great saphenous vein (GSV) was implanted in 100 cases, superficial femoral artery (SFA) was implanted in 34 cases. Early postoperative complications appeared in 16.4% of cases and perioperative mortality (<30 days) was 1.4%. Primary patency at one, three and five years was 59%, 44% and 41%, respectively, while secondary patency was 60%, 45% and 41%, respectively. Primary patency of the SFA allografts was significantly higher than GSV allografts (1 year: SFA: 84% vs. GSV: 51% p = 0,001; 3 years: SFA: 76% vs. GSV: 32% p = 0,001; 5 years: SFA: 71% vs. GSV: 30% p = 0.001). Both primary and secondary patency of SFA allograft implanted in below-knee position were significantly higher than GSV bypasses (p = 0.0006; p = 0.0005, respectively). Limb salvage at one, three and five years following surgery was 74%, 64% and 62%, respectively. Long-term survival was 53% at 5 years.

**Conclusion:**

Allograft implantation is a suitable method for limb salvage in CLTI. The patency of arterial allograft is better than venous allograft patency, especially in below-knee position during infrainguinal allograft bypass surgery.

## Introduction

Infrainguinal autologous conduit bypass surgery is a key revascularization method in chronic limb threatening ischemia (CLTI). The most suitable autologous vascular graft is the ipsilateral single-segment great saphenous vein (GSV), but contralateral GSV, upper limb vein, or small saphenous vein are also suitable for use. Some 15–45% of patients [1–3] require an alternative vascular graft for surgery, due to the lack (earlier coronary or lower limb bypass surgery, varicectomy) or inadequacy (varicosity, insufficient graft diameter or length, structural alterations, earlier thrombophlebitis) of the autologous conduit. An alternative vascular graft may be a prosthetic graft or an allograft.

Following the implantation of prosthetic grafts, lower patency rates were reported, compared to autologous venous bypass, particularly in below-knee bypasses. Alternative autologous veins–such as arm vein conduit–emerges as a potential choice for infrainguinal bypass, however its primary patency is low, 23% at 5 years [4]. The five-year primary patency rate was 38% following below-knee heparin-bonded polytetrafluoroethylene (PTFE) bypasses. [5]. Considering the poor patency rates of synthetic grafts, the high risk of synthetic graft infection due to ulcer or gangrene commonly occurring in CLTI, the use of allografts in limb salvage surgery may be promising.

An allograft is a biological vascular graft, which is explanted during multiple organ donations. In most cases, GSV and/or superficial femoral artery (SFA) are explanted during the donation. Since allografts are classified as biological grafts, using them is more advantageous than prosthetic grafts in situations such as infection, septic condition, ischemic ulcer, and gangrene, since it has appropriate resistance to infection. Moreover, allograft has other advantages, such as very similar compliance and flow dynamics as autologous conduit.

The results following allograft implantation in cases of CLTI are still unclear.

Our aim was to conduct a retrospective analysis of the outcomes of infrainguinal allograft bypass surgery in patients with CLTI over a period of 11 years, and to compare SFA and GSV allografts from the perspectives of graft patency, limb salvage, as well as survival.

## Materials and methods

We conducted a retrospective single-centre observational study of patients with CLTI exposed to infrainguinal allograft bypass surgery between January 2007 and December 2017 with the help of a computerized patient record system and patient follow-up.

Allograft implantation was performed if there was a lack or inadequacy of the ipsilateral or contralateral GSV autologous conduit, taking into account the recommendations for graft selection based on international guidelines [6].

*Our study inclusion criteria*:

- Infrainguinal femoro-popliteal bypass
- Graft material: allograft
- Indication for surgery: CLTI (Fontaine stage III-IV; Rutherford stage 4-5-6)
- TASC C or D femoro-popliteal lesions

*Our study exclusion criteria*:

- Indication for surgery: septic condition, graft infection
- Graft material: composit (autologous vein + allograft)

Patients undergoing allograft implantation due to graft infection were excluded.

During femoro-popliteal allograft bypass surgery, the proximal anastomoses were placed on the common femoral artery, and the distal anastomoses on the above- or below-knee position.

In every case, the implanted allograft was single-segment SFA or GSV. All of the GSV allografts implanted in reverse position. SFA or GSV was choosen by the surgeon intraoperatively depending on the available grafts that matched the required diameter and length. Patients with composite graft (prosthesis+allograft) implantations were excluded.

The implanted allografts were removed from brain-dead donors—with complete donor anonimity—who were found suitable for multi-organ donation conducted by the Hungarian National Blood Transfusion Service, Organ Coordination Office in accordance with the international rules and asepsis guidelines. None of the transplant donors were from a vulnerable population. The consents were not obtained, because by Hungarian law, any person who dies of brain death and fits in the criterias of multi-organ transplantation is considered as a donor and does not require his or her consent in his or her lifetime.

Following explantation by an experienced vascular surgeon, the grafts were placed into a special transport solution [500 ml transport solution: Sodium Chloride 0.9% “Baxter” Intravenous Infusion in Viaflo, (Baxter Hungary, Budapest, Hungary); 4 mg/ml cefazolin (Sandoz GmbH, Kundl, Austria), 0.4 mg/ml fluconazole (Fresenius Kabi Hungary, Budapest, Hungary)] into a triple sterile plastic bag (Set of Transplantation Bags–sterile 80 00 61H, Raguse GmbH, Ascheberg, Germany), kept at 4°C, and delivered to the Allograft Tissue Bank of the Semmelweis University, Department of Vascular and Endovascular Surgery, Budapest, Hungary, where they were prepared carefully and frozen within 24 hours [7].

During graft conservation, the cryopreservation was performed in a clean room classified “A” with a background classified “B” used laminar air flow system and the grafts were frozen in a cryopreservation solution (500 ml cryopreservation solution (Ringer Fresenius, Fresenius Kabi Deutschland GmbH, Bad Homburg, Germany) containing 20 v/v% dimethyl sulfoxide (Molar Chemicals Kft., Halásztelek, Hungary), 4 mg/ml cefazolin (Sandoz GmbH, Kundl, Austria) and 0.4 mg/ml fluconazole (Fresenius Kabi Hungary, Budapest, Hungary), and stored at -80°C. At the beginning of the surgery, the graft to be implanted was thawed in a water bath at 20–25°C and then implanted after preparation. We did not consider blood type and we did not administer any immunosuppressive drugs.

Regarding limb salvage, only major amputations at the below-knee or above-knee levels were taken into consideration. Graft patency were investigated only in cases where the follow-up time was complete. Primary and secondary patency were defined according to the Society for Vascular Surgery (SVS) definitions [8].

Long term patient follow-up was performed by the operating surgeon at 1, 6, and 12 months and then annually after the surgery. If there was suspicion for stenosis or occlusion of the allograft during the clinical examination, duplex US was performed.

Graft infection was definied according to Szilagyi classification [9].

Statistical analysis of the data was performed using the GraphPad Prism software (GraphPad Software, Inc., La Jolla, CA, USA). Student t-test, Fisher’s method, multivariable Cox-regression and Kaplan-Meier analysis were used. We checked the distribution of the variables by using the Shapiro-Wilk normality test and presented continuous data as mean with standard deviation or median with interquartile ranges, as appropriate. Categorical data were described as event numbers with percentages. A 95% confidence interval was considered to be statistically significant (p <0.05).

During the course of our study, data were handled in accordance with the valid legal regulations and the work was carried out with the permission of the Regional, Institutional Scientific and Research Ethics Committee of Semmelweis University (#SE TUKEB 132/2015). All data handling and data processing met the requirements of Act LXIII of 1992 on the Protection of Personal Data and Disclosure of Data of Public Interest and Act XLVII of 1997 on the Processing and Protection of Medical and Other Related Personal Data. Before surgery, all patients received adequate information about both the surgery and the use of their personal data in medical research. Written and oral medical information was given to all patients. Written informed consent was obtained from all patients participating in the study, in accordance with the approved Institutional Review Board (IRB) protocols.

## Results

### Patient characteristics, operative details

Between January 2007 and December 2017 a total of 134 cases of infrainguinal allograft implantations due to CLTI were performed. The mean age of the patients was 66.4 ± 9.9 years, with the majority of them being males (91 patients, 67.9%).

GSVs were implanted in 100 cases (74.6%), SFAs in 34 cases (25.4%).

Grafts were implanted at above-knee position in 35 cases (26.1%) and at below-knee position (popliteal, tibioperoneal trunk) in 99 cases (73.9%).

The main cardiovascular risk factors detailed in Table 1.

**Table 1:** Demographic data and comorbidities.

*Features*	*Mean±SD*	
*Age (years)*	66.4±9.9	
	**Cases (N)**	**%**
*Male*	91	67.9
***Comorbidities***		
*Smoking*	107	79.8
*Hypertension*	121	90.2
*Hyperlipidemia*	106	79.1
*Coronary diseases*	49	36.5
*Diabetes*	56	41.7
*Stroke*	16	11.9
*COPD*	14	10.4
*Renal failure*	2	1.4

### Postoperative outcomes

The mean length of hospital stay was 13.6 ± 5.6 days.

Early postoperative complications (<30 days) occurred in 16.4% of the cases, among which significant cardiovascular complications occurred in 2 cases (1.4%): stroke in one patient and acute myocardial infarction in another patient. Hematoma and wound infection requiring reoperation occured in 4 (2.9%) and 6 (4.4%) cases, respectively. (Table 2).

**Table 2:** Early postoperative complications.

*Complication*	*Cases (N)*	*%*
*Occlusion*	10	7.4
*Hematoma*	4	2.9
*Wound infection*	6	4.4
*Stroke*	1	0,7
*AMI*	1	0.7

Reoperations (open surgery) were performed in 26.1% of cases. (Table 3) Graft infections developed in 2 cases (1.4%). Both graft infections were Grade III infections according tot he Szilagyi classification. In one case, graft ligation was performed due to septic bleeding followed by combined antibiotic therapy. In the other case, wound exploration, lavage and necrectomy were performed, followed by combined antibiotic therapy.

**Table 3:** Reoperations.

*Early (<30 days) (n = 14)*	*Late (>30 days) (n = 21)*
Graft occlusion (n = 10)	Graft occlusion (n = 18)
Graft lesion–bleeding, hematoma (n = 3)	Graft dilatation (n = 1)
Wound infection (n = 1)	Proximal anastomosis pseudoaneurysm (n = 1)
	Graft infection (n = 1)

Neither early postoperative complications nor reoperations were significantly affected by homograft type and the location of the distal anastomosis (early postoperative complications vs. location of the distal anastomosis: p = 0.21; vs. allograft type: p = 0.14; early reoperations vs. location of the distal anastomosis: p = 0.55; vs. allograft type: p = 0.68; late reoperations vs.location of the distal anastomosis: p = 0.34; vs. allograft type: p = 0.97.

Structural changes (aneurysm or dilatation) occurring in the graft, which were distant from the anastomosis, were described in 2 cases (1.4%). In one case, the entire segment of the allograft was dilated, resulting in prosthetic graft replacement. In the other case, an aneurysm (d = 22mm) formed on the distal segment of the allograft, which was occluded and no surgery was performed.

**Fig 1:**
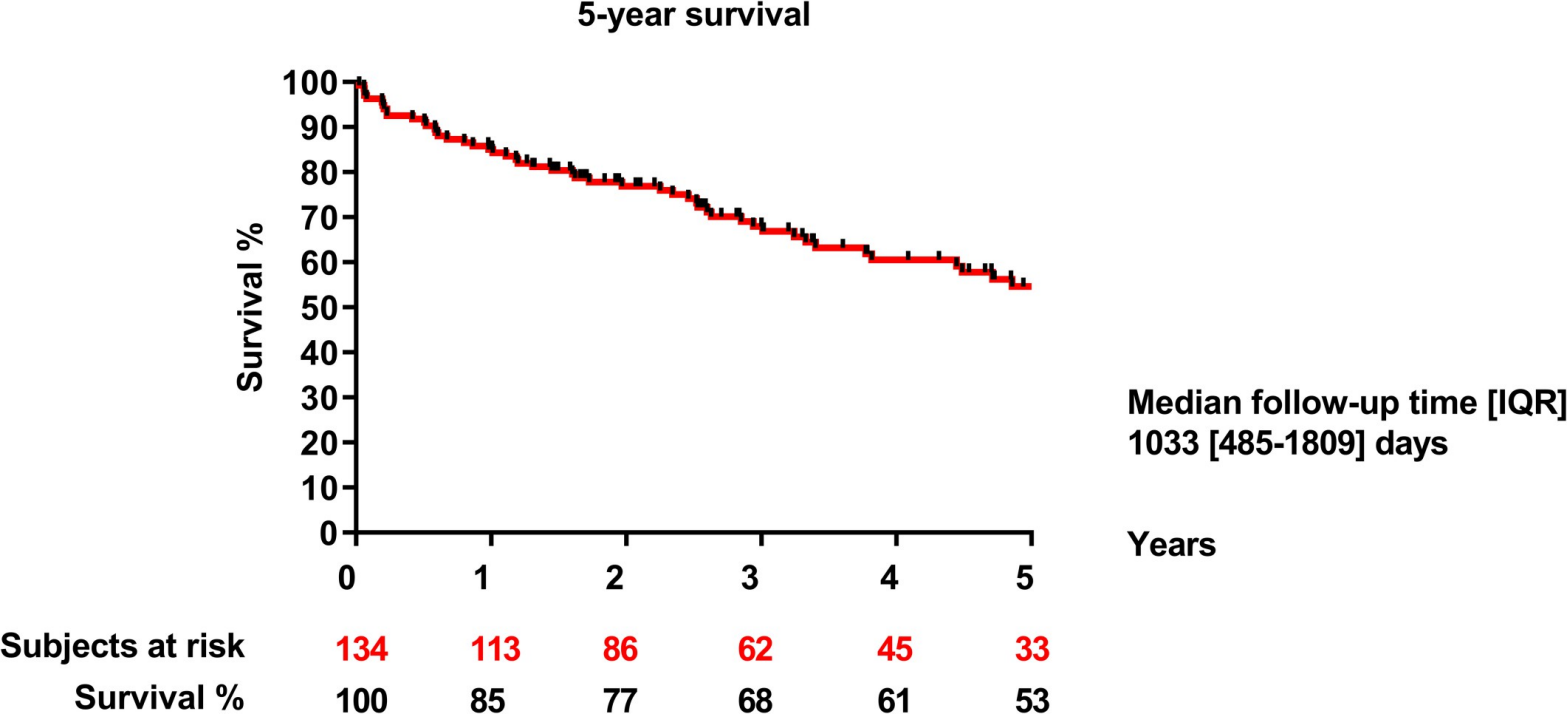
5-year survival.

The perioperative mortality rate was 1.4% (2 cases). Long-term survival at one, three and five years after surgery was 85%, 68% and 53%, respectively (Fig 1).

### Graft patency, limb salvage

**Fig 2:**
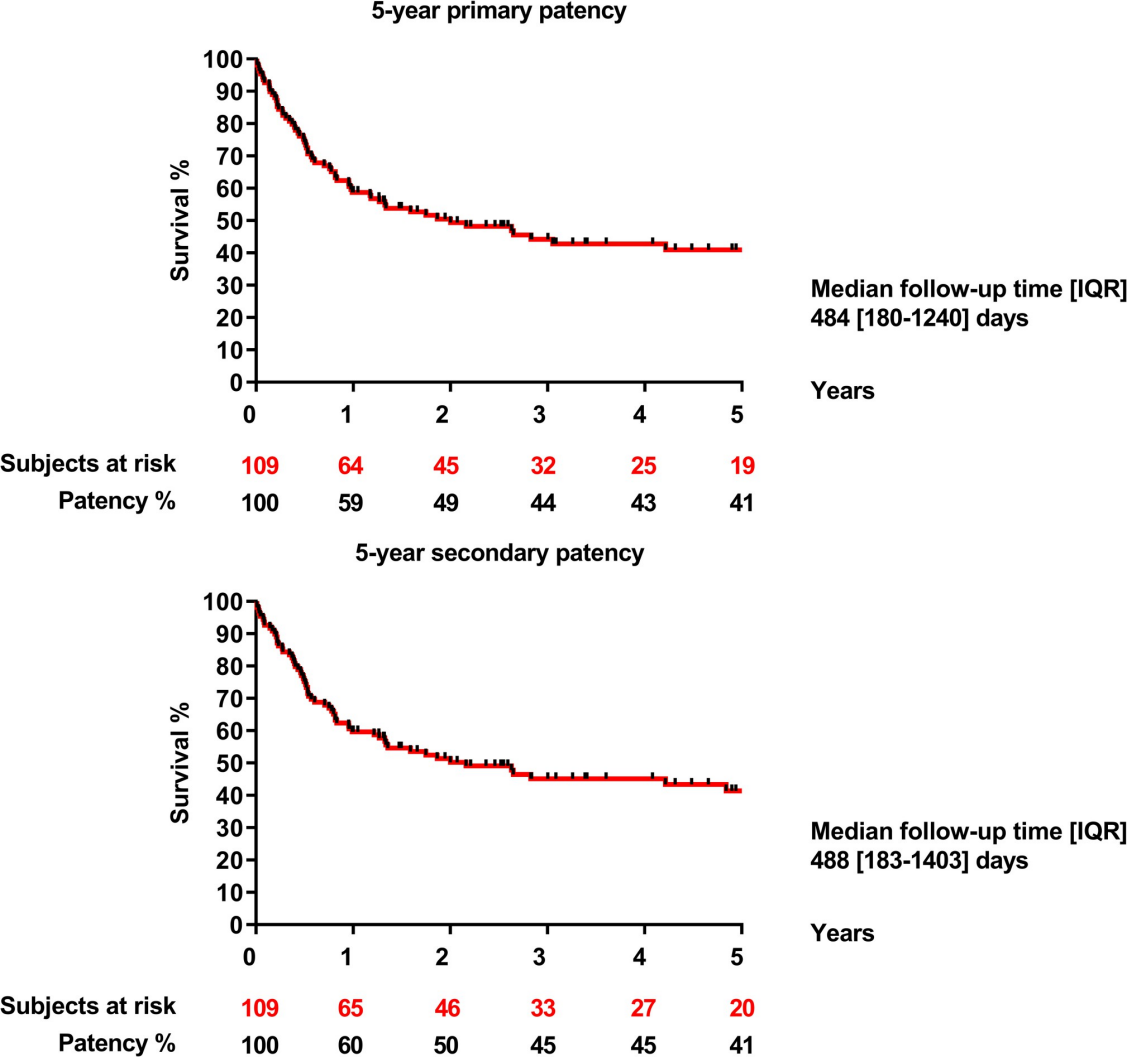
5-year primary and secondary patency.

**Fig 3:**
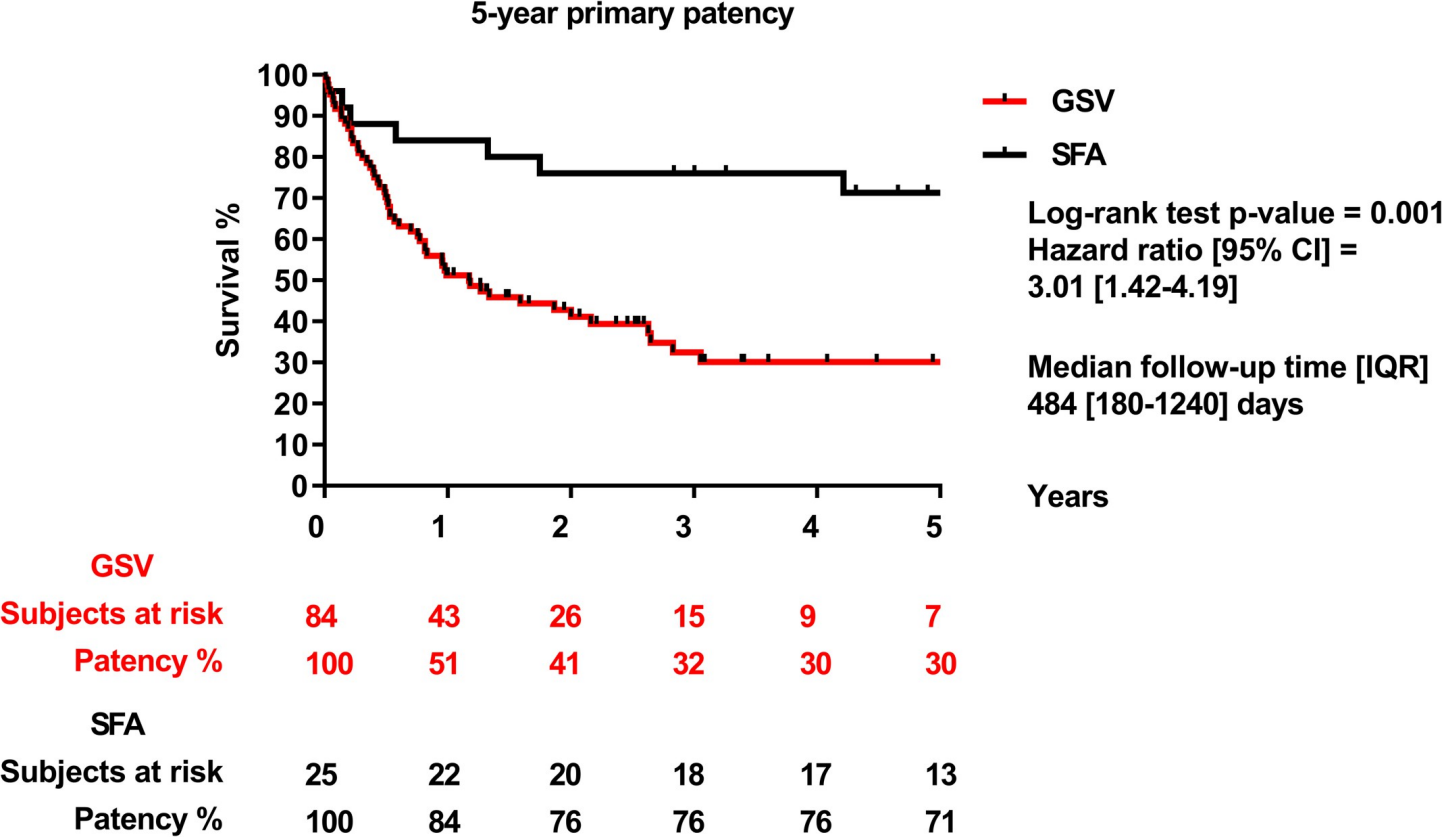
5-year primary patency of GSV (great saphenous vein) and SFA (superficial femoral artery).

**Fig 4:**
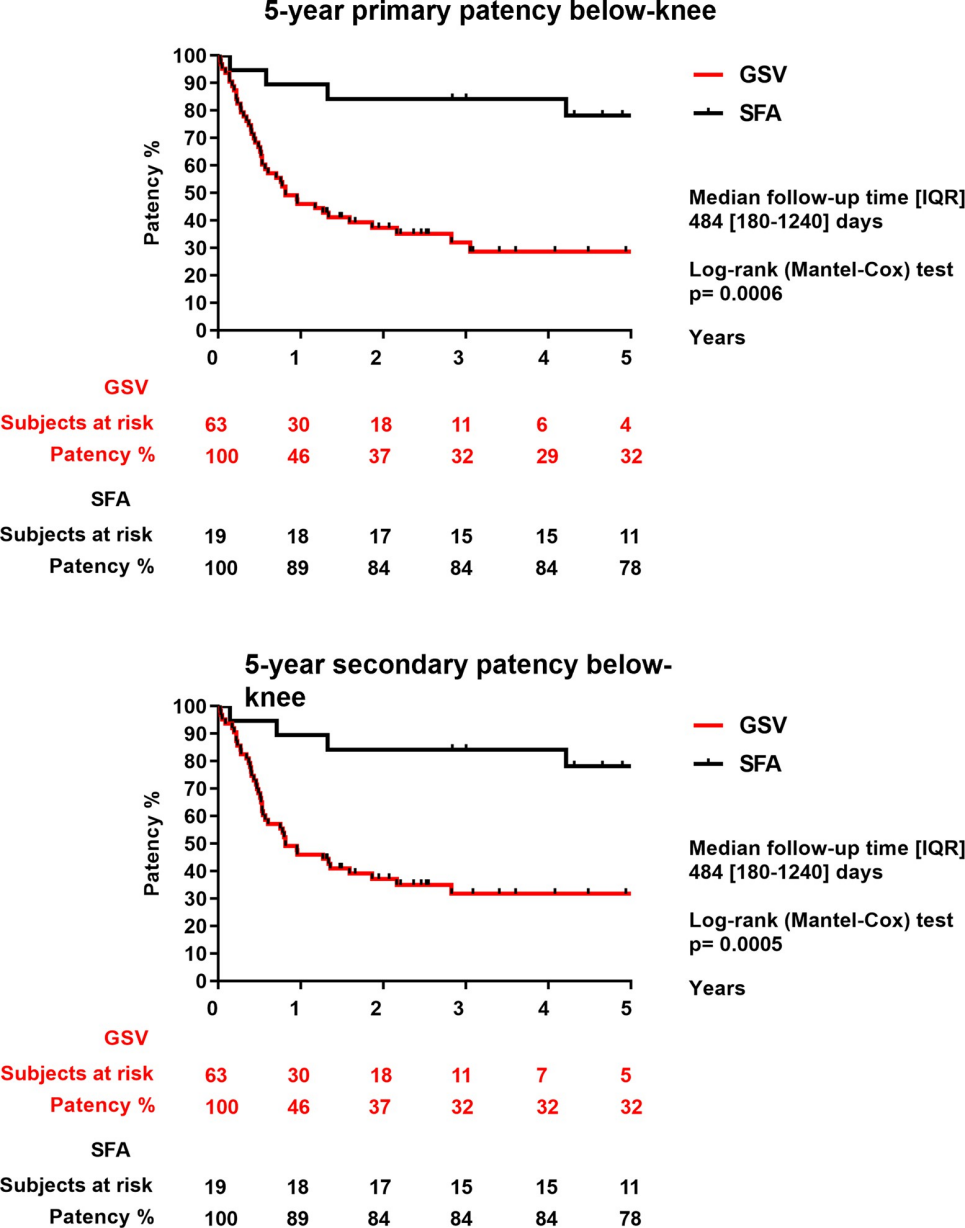
5-year primary and secondary patency of GSV (great saphenous vein) and SFA (superficial femoral artery) implanted in below-knee position.

Primary patency of the entire group was 59% after one year, 44% after three years, and 41% after 5 years. Secondary patency of the entire group after 1, 3 and 5 years was 60%, 45% and 41%, respectively (Fig 2). SFA allograft patency rate was significantly higher compared to the GSV allograft patency rate (1 year: SFA: 84% vs. GSV: 51% p = 0.001; 3 years: SFA: 76% vs. GSV: 32% p = 0.001; 5 years: SFA: 71% vs. GSV: 30% p = 0.001) (Fig 3). Both primary and secondary patency rate of SFA allografts were significantly higher than GSV allografts in the below-knee position (primary patency at 1 year: SFA: 89% vs. GSV: 46% p = 0.0006; 3 years: SFA: 84% vs. GSV: 32% p = 0.0006; 5 years: SFA: 78% vs. GSV: 32% p = 0.0006). The secondary patency results at 1 year: SFA: 89% vs. GSV: 46% p = 0.0005; 3 years: SFA: 84% vs. GSV: 32% p = 0.0005; 5 years: SFA: 78% vs. GSV: 32% p = 0.0005) (Fig 4). Neither primary, nor secondary patency rate of SFA allografts were significanty different from GSV allografts implanted at the above-knee position (p = 0.78; p = 0.79).

**Fig 5:**
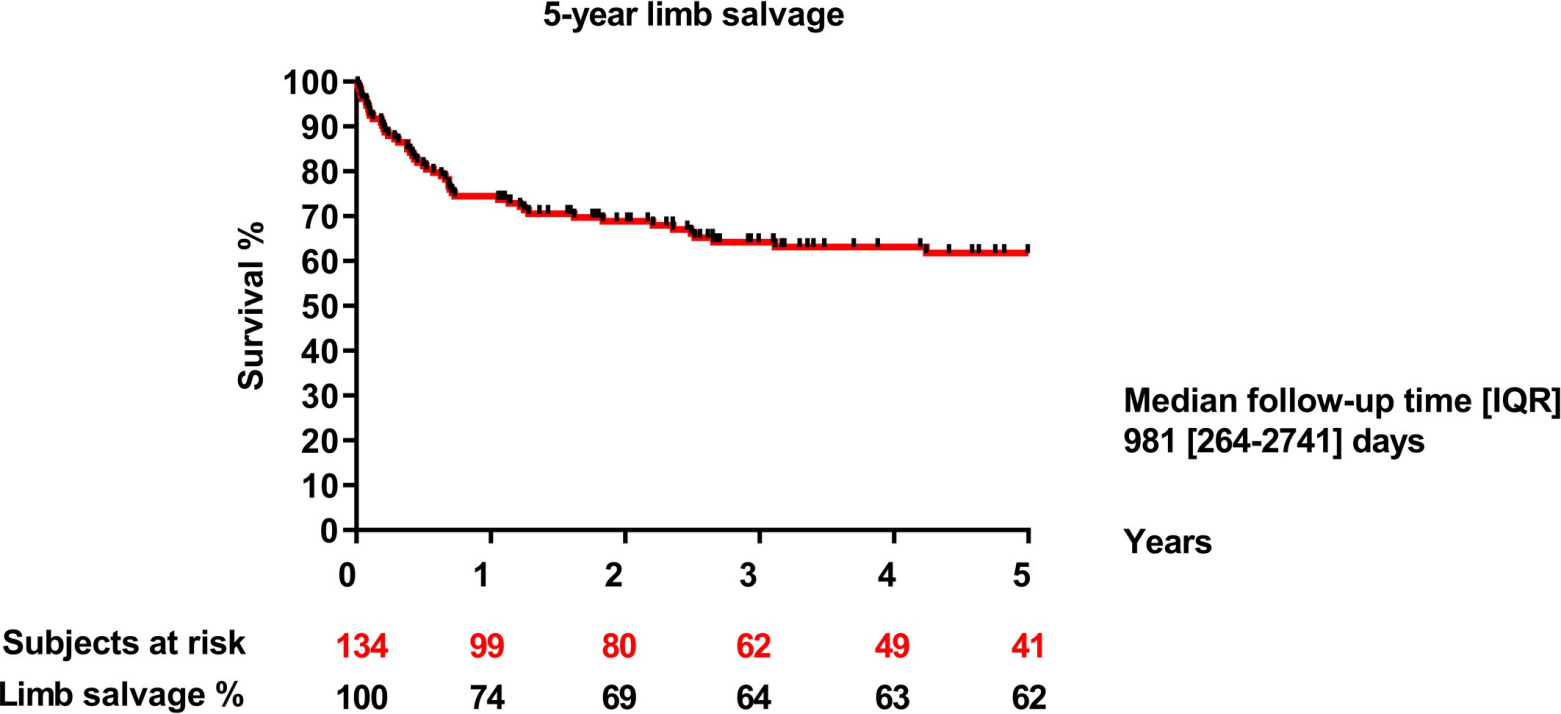
5-year limb salvage.

Limb salvage rates at one, three and five years after surgery were 74%, 64% and 62%, respectively (Fig 5). Neither the allograft type (3 years: SFA: 35% vs. GSV: 38%; p = 0.679) nor the location of the distal anastomosis (3 years: below-knee: 38% vs. above-knee: 40%; p = 0.907) affected limb salvage significantly.

## Discussion

Even nowadays there are a significant number of patients with CLTI who need to undergo vascular surgery. The estimated annual incidence is 220–3500 cases per 1 million people with a prevalence of 1% in the adult population [10,11]. Twenty percent of patients require amputation and 25% die within a year after the onset of critical limb ischemia [12]. The best method of revascularization, meaning either open or endovascular intervention, is key to limb salvage.

In case of open surgery, the first method of choice is autologous GSV femoro-popliteal bypass, which has the most favorable patency compared to alternative graft types [13,14]. In the absence of an autologous conduit or in case of unsuitability, an alternative vascular graft has to be chosen, which, in most cases, would be a prosthetic graft or an allograft.

Prosthetic bypass grafts are not ideal for patients with Fontaine IV gangrene or ulcer due to the potential risk of infection and in addition such grafts have poor patency rates [15]. Uhl et al. [16] investigated the patency of below-knee femoro-popliteal bypass surgery with autologous vein compared to heparin-bound PTFE grafts, and found that the use of an autologous vein continues to be the first choice for revascularization surgery below the inguinal ligament.

In the absence of autologous conduit allograft is an alternative option for a vascular graft. Most ot the available literature on femoro-popliteal allograft bypass surgeries involve the use of allografts for graft infection. There are only a few data available on the results of allograft implantations in CLTI.

In this retrospective analysis, we studied the data of patients who underwent femoro-popliteal allograft bypass surgery due to CLTI, and we compared the results of the two most commonly used allograft types, SFA and GSV, in terms of graft patency, limb salvage, and survival. There are relatively few centers in Europe using allografts and most of their earlier reports present a small number of cases.

The majority of the implanted allografts were GSVs (74.6%), SFAs were implanted in 25.4% of the cases. There are hardly any reports that compare the results of venous and arterial allografts. The vast majority of publications report the results of venous allografts [17–21], with fewer studies presenting the results of arterial allograft implantations [22,23].

The possibility of graft degeneration is important when considering the use of an allograft, which may question its use as opposed to prosthetic graft. In our study, graft degeneration was detected in only 2 out of the 134 cases. Masmejan et al. [22] reported graft degeneration in one of 42 cases, and Albertini et al. [24] found 4 cases among 165 bypasses. Our results support the literature data, according to which there are a negligible number of degenerations in case of allografts. Careful explantation of grafts from the donor, cautious tissue conservation and preoperative preparation, as well as strict adherence to basic vascular surgical principles are important issues in relation to the prevention of graft degeneration.

One year after the implantation, the allograft showed a primary patency rate of 59%, and 41% at five years. Secondary patency rates did not differ significantly from the primary patency rates. The reason for the high number of occlusions was the high occlusion rate of venous grafts, we therefore examined the patency of GSV and SFA allografts. One, three, and five years after surgery, the patency of SFA allografts was significantly higher as compared to the GSV allografts. Masmejan et al. [22] studied 42 cases of arterial allografts during a 10-year period, finding graft patency rates of 60% at 1 year and 26% at 5 years. Lejay et al. [25] reported a primary patency rate of 59% at 5 years when studying 28 arterial allograft implantations. Investigating the outcomes of infrainguinal allograft bypass surgeries after one year, O’Banion et al. [18] reported a primary patency rate of 35%, Ziza et al. [20] of 47% and Randon et al. [17] of 56%. Our results are in accordance with the literature findings, and are in line with previous studies on the graft patencies of venous and arterial allograft types. Better patency rates of SFA allograft owing to the below-knee patency results, where SFA allograft shows significantly higher patency rates than GSV allograft.

The higher occlusion rate of venous allografts may be explained by the smaller vessel diameter, the weaker structure of the vessel wall–thus being liable to degeneration–and the more vulnerable intimal layer, making it prone to thrombosis. In their study of 1404 patients who underwent lower limb bypass surgery due to CLTI, Schanzer et al. [13] found that grafts with smaller vessel diameters had negative effects on subsequent graft patencies.

Arterial allografts may be anatomically and physiologically more suitable than venous grafts, owing to the more resistant mechanical properties when exposed to arterial blood flow and blood pressure. Walden et al. [26] denoted that matching graft and artery elastic properties resulted in higher patency rates. According to Pukacki et al. [27], cryopreservation maintains the elastic properties of arterial allografts, which is mostly the same as the elasticity of the recipient vessel.

The site of distal anastomosis–above-knee or below-knee–may have a considerable impact on graft patency. Earlier studies proved that below-knee distal anastomosis showed lower patency rates than in case of proximal bypass [13,28]. In our analysis, although below-knee bypasses showed somewhat lower patency rates, we could not detect significant differences between the patencies of above- and below-knee anastomoses. O’Banion et al. [18] reported similar results. Better patency rates have been described in allograft bypasses for the superficial femoral artery or the above-knee section of the popliteal artery than for the below-knee section of the popliteal artery or for distal anastomosis located at the crural arteries, however, no significant differences could be confirmed.

In our study, the allograft occlusion rate was found to be high, but limb salvage showed an acceptable rate. Five years after surgery, 62% of the operated limbs were still viable. Similarly to our results, Randon et al. [17] reported a limb salvage rate of 65% at 5 years with a primary patency rate of 11.1% and a secondary patency rate of 38.5%. The reason for the acceptable long-term amputation rate is that during bypass graft patency, the distal limb tissues in even Fontaine stage IV patients received a sufficient amount of blood flow for the ischemic ulcer or gangrene to heal. In the event of early graft occlusion, wound healing contributes to long-term limb survival.

The low rate of long-term survival– 53% at 5 years–is in accordance with the literature data according to which patients with CLTI have a 5 year survival rate of 50–60% [29,30]. The high mortality rate is not directly caused by the peripheral vascular disease or surgery, but rather by comorbidities and risk factors related to diffuse cardiovascular disease with negative effects on survival.

Our study has its limitations, which may influence the obtained results and restrict actual prediction outcomes. In this retrospective nonrandomized study, all pre-, intra-, and postoperative assessments, methods and decisions were at the discretion of the operating surgeon, we did not follow a specific, strict protocol. Implanted arterial and venous allografts were not randomized due to the nature of the retrospective study and the limited availability of allografts.

## Conclusions

In our retrospective analysis involving a large number of cases, we analysed the results of infrainguinal allograft bypass surgery in patients with CLTI.

Allograft implantation is known to be a good alternative to prosthetic graft, showing promising results in case of risk of infection, which is a common morbidity after bypass procedures for CLTI.

Our results showed the patency rates to be low, but acceptable. Our study is the first to report that SFA allograft is a better option than GSV at below-knee position in patients with CLTI, since both primary and secondary patency of SFA graft was found to be significantly higher.

Further prospective randomized studies are needed in order to evaluate allograft bypass surgeries performed in case of CLTI.

Our results call attention to the fact that during femoro-popliteal bypass surgery in patients with CLTI, allograft implantation–often as a last resort for limb salvage–seems to be a suitable method for revascularization in the absence of an autologous conduit.

## Supporting information

S1 Database (XLSX)
